# Effectiveness of bilateral tubotubal anastomosis in a large outpatient population

**DOI:** 10.1093/humrep/dew038

**Published:** 2016-03-14

**Authors:** Gary S. Berger, John M. Thorp, Mark A. Weaver

**Affiliations:** 1 Department of Obstetrics and Gynecology, University of North Carolina at Chapel Hill School of Medicine, 3025 Old Clinic Building, Campus Box 7570, Chapel Hill, NC 27599, USA; 2 Departments of Medicine and Biostatistics, University of North Carolina at Chapel Hill, 258 Brinkhouse-Bullitt, Campus Box 7064, Chapel Hill, NC 27599, USA

**Keywords:** bilateral tubotubal anastomosis, outpatient, female surgical sterilization, sterilization reversal, pregnancy

## Abstract

**STUDY QUESTION:**

Is bilateral tubotubal anastomosis a successful treatment in an outpatient patient population?

**SUMMARY ANSWER:**

For women wanting children after tubal sterilization, bilateral tubotubal anastomosis is an effective outpatient treatment.

**WHAT IS KNOWN ALREADY:**

With the current emphasis in reproductive medicine on high technology procedures, the effectiveness of female surgical sterilization reversal is often overlooked. Previous clinical studies of tubal sterilization reversal have been mostly retrospective analyses of small patient populations.

**STUDY DESIGN, SIZE, DURATION:**

A cohort of women who underwent outpatient bilateral tubotubal anastomosis from January 2000 to June 2013 was followed prospectively until December 2014 to determine the proportions of women undergoing the procedure who became pregnant and who had live births. Data were collected at the time of pregnancy. Differences in pregnancy rates and live birth rates associated with age, race and sterilization method were evaluated.

**PARTICIPANTS/MATERIALS, SETTING, METHODS:**

A total of 6692 women, aged 20–51 years, underwent outpatient bilateral tubotubal anastomosis.

**MAIN RESULTS AND THE ROLE OF CHANCE:**

The crude overall pregnancy rate was 69%. The crude overall birth rate was 35%. Results varied according to age at sterilization reversal and the method of sterilization. Women under 30 years of age at reversal of ring/clip sterilizations had an 88% pregnancy rate and 62% birth rate. Pregnancy and birth rates declined as age increased at sterilization reversal. Coagulation sterilization reversals resulted in the lowest rates of pregnancies and births. Ligation/resection reversals had intermediate success rates.

**LIMITATIONS, REASONS FOR CAUTION:**

Limitations of our study include probable underreporting of pregnancies based on patient-initiated reports; possible errors in the reporting of pregnancies or early miscarriages that may have been based solely on home pregnancy tests; and probable over-reporting of the diagnosis of ectopic pregnancies. We identified age and sterilization method as being associated with subsequent pregnancy, however, in order to be considered predictive, the associations would need to be validated in an independent second prospectively studied group of representative patients. Finally, we also included patients in the study population who had additional surgical procedures performed at the time of tubotubal anastomosis (e.g. uterine myomectomy, fimbrioplasty, ovarian cystectomy and adhesiolysis), factors that could result in differences in pregnancy statistics in our study versus other patient populations.

**WIDER IMPLICATIONS OF THE FINDINGS:**

The results of this study can help inform patients and clinicians about this low technology alternative to IVF.

**STUDY FUNDING/COMPETING INTEREST(S):**

None.

**TRIAL REGISTRATION NUMBER:**

N/A.

## Introduction

As divorce and remarriage have become more common in recent decades, increasing numbers of women want to have children after a tubal sterilization. During this time, reproductive endocrinology and infertility specialists have largely dismissed sterilization reversal surgery in favor of assisted reproductive technology. This change in practice occurred without a supporting evidence base. The predilection for highly technological treatments that outrun evidence of superiority, accompanied by the phasing out of useful lower technology treatments, may not be in the best interest of patients. With this as the background, we studied the effectiveness of bilateral tubotubal anastomosis for restoring fertility after tubal sterilization in a large patient population.

This report answers three questions patients ask most often about sterilization reversal surgery:

How likely am I to become pregnant?; If I become pregnant, what is the risk of having an ectopic pregnancy or miscarriage? and Overall, what is my chance of having a baby?

## Methods

### Study design

This is the initial report from a prospective observational study of 9669 women who had tubal reparative surgeries. The surgeries were performed in an outpatient surgical center in Chapel Hill, NC, USA, from January 2000 to June 2013.

### Study participants

Among all women in the tubal surgery database, 6692 underwent bilateral tubotubal anastomosis in an effort to conceive after tubal sterilization with follow-up data recorded. These women comprise the study population for this analysis.

### Surgical method

Tubotubal anastomosis was defined as the joining of any two tubal segments including: interstitial, isthmic, ampullary, infundibular or fimbrial segments. Surgery was performed adhering to microsurgical principles through mini-laparotomy incisions with 4× loupe magnification. Pre-emptive analgesia and local anesthesia supplemented general anesthesia. Mean operating time was 59 min with a standard deviation (SD) of 12.0 min.

Before surgery, acetaminophen 975 mg and ibuprofen 800 mg were administered by mouth for preventive analgesia. Other preoperative medications included oral promethazine 12.5 mg for nausea control and clonidine 0.05 mg for mild sedation.

Local anesthetic solution consisting of 0.25% bupivacaine with 1:200 000 epinephrine was injected into the skin prior to making a transverse suprapubic incision. Subcutaneous tissue and fat were incised with monopolar electrodissection. Local aesthetic solution was injected into the rectus fascia that was also incised electrosurgically. The fascia was dissected from the underlying rectus abdominis and pyramidalis muscles that were separated in the midline. The peritoneum was picked up and entered with sharp dissection. Finger retraction was used to exposure the pelvic organs.

The tubal segments were elevated with finger compression as transections were performed across adjacent tubal edges with Iris scissors. When significant discrepancies were present between the tubal segments, the narrower segment was incised along its antimesenteric border to reduce the diameter differences at the anastomosis sites.

The tubal lumen was splinted with #1 monofilament polypropylene, passing the stent proximally into the endometrial cavity and distally through the fimbrial end. This technique ensured patency from the uterine cavity throughout the length of the tube; it also stabilized the tube during the anastomosis. A retention suture was placed in the mesosalpinx with 3-0 absorbable monofilament glyconate. End-to-end tubotubal anastomosis was performed incorporating the muscularis and serosa in a single layer with interrupted sutures of 6-0 non-absorbable monofilament polypropylene/polyethylene. Care was taken to avoid the endothelial layer. The stent was withdrawn from the fimbrial end of the tube. Throughout the operation, the tubal segments and other pelvic organs were irrigated with warm heparinized Lactated Ringer's solution and microsurgical electrocautery was used for meticulous hemostasis. After ensuring the irrigating solution was completely clear of any hemosiderin pigment, the rectus muscles were approximated in the midline with interrupted 3-0 absorbable glycolide sutures. These sites were infiltrated with local anesthetic solution. The rectus fascia was approximated with #0 polydioxanone interrupted sutures. These suture sites were also infiltrated with local anesthetic solution. Camper's fascia and Scarpa's fascia were approximated with 3-0 glycolide interrupted sutures. A running suture of non-pigmented 4-0 glycolide was used to close the reticular layer of the skin.

### Data collection and analysis

We reviewed patients' sterilization operative reports before reversal surgery to determine sterilization method. At 1 year after surgery, nurses initiated email and telephone inquiries using standardized forms to record pregnancy information. Up to three email inquiries and two telephone calls were made to each patient in efforts to obtain information about pregnancy status. Additional information about pregnancies after surgery came from patient-initiated reports submitted through website forms, email, postal mail or telephone at any time throughout the duration of the study. Nurses recorded all data contemporaneously in an electronic database.

We used survival analytic methods to assess associations between time until first pregnancy and time until first live birth with sterilization method and age at time of sterilization reversal. For each patient, we defined time 0 as the date of surgery. For those who became pregnant, we estimated the date of conception as follows: for women who reported a last menstrual period date (LMP), we used LMP + 14 days; for women without an LMP but who reported any positive pregnancy tests, we used the date of the first positive test; for women without reported LMP and without positive pregnancy tests, we used the date of birth minus 38 weeks; for all other women, we used the midpoint between the date of surgery and the date that follow-up data were obtained. For women who experienced a live birth, we used the baby's date of birth when available; otherwise, we used the estimated date of conception plus 38 weeks. As described above, active follow-up ended 1 year post-surgery with voluntary reporting thereafter. Thus, to mitigate the potential for informative censoring, we took a conservative approach; for women who never reported experiencing the event of interest (pregnancy or live birth) and whose last contact was within 3 years post-surgery, we assumed that they did not experience the event during the 3 years. Therefore, estimated cumulative event probabilities should be interpreted as approximate lower bounds. We compared time with first pregnancy or first live birth across patient characteristics (age at surgery and sterilization method) separately using log-rank tests. We used Kaplan–Meier estimators to estimate the cumulative incidences of pregnancy or live birth during the 3 years post-surgery. Analyses were conducted using SAS, version 9.3 (SAS Institute, Cary, NC, USA). A value of *P* < 0.05 was considered significant.

The University of North Carolina at Chapel Hill IRB and Office of Human Research Ethics gave this study exempt status (IRB Number 14-1783) as a quality improvement study, meaning that written consent was not required.

## Results

### The study population

The ages of the 6692 women at the time of tubotubal anastomosis ranged from 20 to 51 years. The mean age was 34.6 years with a SD of 4.5. The age distribution was: less than 30 years (13%), 30–34 years (37%), 35–39 years (36%) and 40 years or older (14%). The distribution by race/ethnicity was: white (77%), black (12%), Hispanic (7%) and other or not stated (4%). The most common method of previous sterilization was tubal ligation/resection (40%). Mechanical methods (tubal rings or clips) accounted for 25% of the population and coagulation methods for 26%. Nine percent of the women had undergone other sterilization techniques. These included hysteroscopic tubal occlusive sterilizations, combinations of sterilization methods or unknown procedures.

### Pregnancy incidence

In total, 4633 (69%) women reportedly became pregnant during follow-up (Table I). Pregnancy percentages ranged from 82% for women <30 years of age to 38% for women age 40 or older at the time of tubal repair (*P* < 0.001).

**Table I:** Patient characteristics according to ever reporting a pregnancy during 14 years of follow-up.

Characteristic	Total *n*	Ever pregnant after tubotubal anastomosis		*P*-Value*
		Yes, *n* (%)	No, *n* (%)	
Age at surgery (years)				<0.001
<30	899	737 (82%)	162 (18%)	
30–34	2451	1907 (78%)	544 (22%)	
35–39	2378	1625 (68%)	753 (32%)	
≥40	964	364 (38%)	600 (62%)	
Sterilization method				<0.001
Ring/clip	1661	1267 (76%)	394 (24%)	
Ligation/resection	2688	1838 (68%)	850 (32%)	
Coagulation	1762	1181 (67%)	581 (33%)	
Other/NS^†^	581	347 (60%)	234 (40%)	
Total	6692	4633 (69%)	2059 (31%)	

^†^Includes various combinations of methods (25%) and unknown or not specified (NS).

*Log-rank *P*-value comparing time until first pregnancy across characteristic groups.

**Figure 1:**
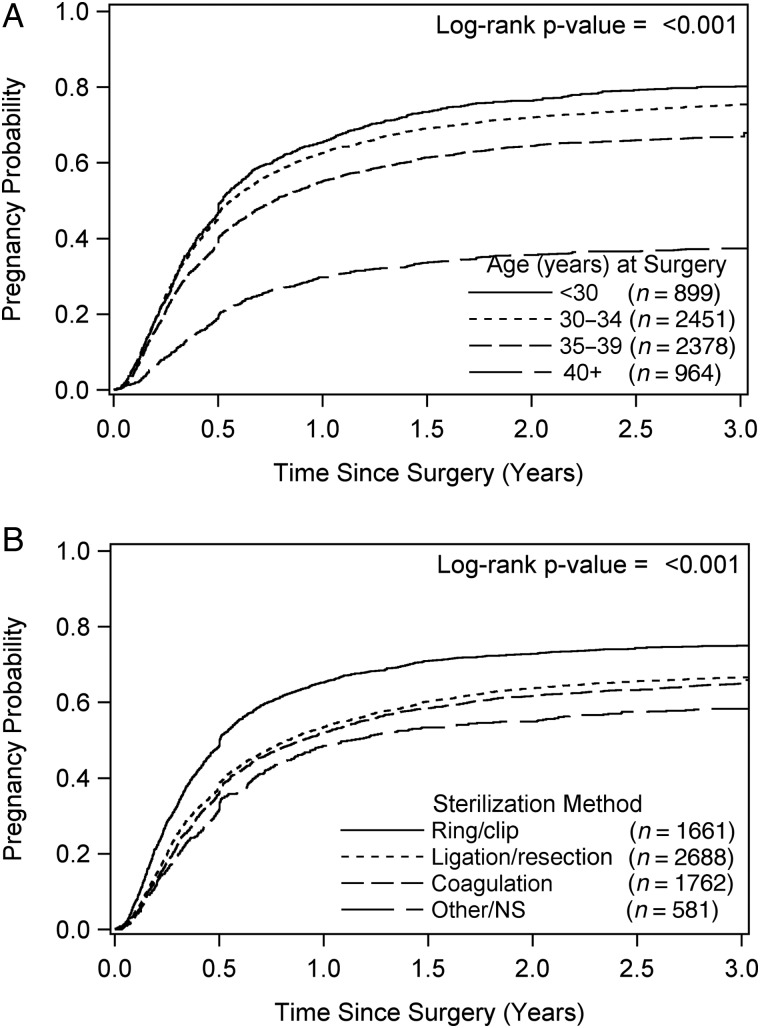
Kaplan–Meier estimates of cumulative pregnancy probabilities over 3 years by (**A**) age at surgery group and (**B**) sterilization method. NS, not specified.

Pregnancy percentages were highest for women after reversal of ring/clip sterilizations (76%), followed in descending order by those with ligation/resection (68%) and coagulation (67%) procedures (*P* < 0.001). The overall cumulative incidence of pregnancy at 6 months was 41%, 95% confidence interval [40%, 42%] and at 12 months was 58% [57%, 59%]. Estimated cumulative incidences over 3 years, by age at sterilization reversal and by sterilization method, are shown in Fig. 1.

We further investigated the pregnancy incidence in subpopulations defined jointly by age group and sterilization method (Table II). Women younger than 35 years of age who had ring/clip procedures experienced the highest 12-month cumulative incidence of pregnancy (73–77%), while for women aged 40 years or older the 12-month cumulative incidence was lower than 50% regardless of sterilization method.

**Table II:** Pregnancy incidence by age and sterilization method.

Sterilization method	Age (years)	Number of women	Became pregnant, *n* (%)	Cumulative incidence of pregnancy	
				6-Month (%)	12-Month (%)
Ring/clip	<30	267	233 (88%)	60	77
	30–34	619	513 (83%)	55	73
	35–39	559	419 (75%)	49	66
	≥40	216	99 (46%)	29	44
Ligation/resection	<30	359	289 (81%)	45	63
	30–34	1017	777 (76%)	45	61
	35–39	952	643 (68%)	39	55
	≥40	360	129 (36%)	18	33
Coagulation	<30	216	170 (79%)	43	62
	30–34	648	488 (75%)	43	61
	35–39	654	435 (67%)	38	57
	≥40	244	88 (36%)	15	26
Other/NS	<30	57	42 (74%)	51	61
	30–34	167	129 (77%)	43	66
	35–39	213	128 (60%)	32	50
	≥40	144	48 (33%)	18	29

### Pregnancy outcomes

The 6692 women in this study reported a total of 7275 pregnancies. Over one-third (37%) of women who ever became pregnant during follow-up reported more than one pregnancy. The range was 1–9 pregnancies, with a mean of 1.5 and SD of 0.9. The distribution of outcomes for the entire group of pregnancies was (Table III): birth (39%), ongoing pregnancy (14%), miscarriage (34%) and ectopic pregnancy (13%).

**Table III:** Distribution of pregnancy outcomes* by age and sterilization method.

Characteristic	Birth, *n* (%)	Ongoing, *n* (%)	Miscarriage, *n* (%)	Ectopic, *n* (%)	Total reported pregnancies
Age at surgery (years)					
<30	535 (43%)	177 (14%)	379 (31%)	143 (12%)	1234
30–34	1270 (41%)	432 (14%)	965 (31%)	422 (14%)	3089
35–39	890 (36%)	346 (14%)	895 (36%)	336 (14%)	2467
≥40	121 (25%)	77 (16%)	232 (48%)	55 (11%)	485
Sterilization method					
Ring/clip	993 (50%)	260 (13%)	583 (29%)	155 (8%)	1191
Ligation/resection	1047 (36%)	442 (15%)	1009 (35%)	417 (14%)	2915
Coagulation	592 (32%)	248 (13%)	703 (38%)	313 (17%)	1856
Other/NS	184 (36%)	82 (16%)	176 (34%)	71 (14%)	513
Total	2816 (39%)	1032 (14%)	2471 (34%)	956 (13%)	7275

^*^Some women reported multiple pregnancies.

Pregnancies among younger women were more likely to result in birth than among older women. This was related to a higher rate of miscarriage among older women. Women with tubal clip or ring sterilizations had the highest proportion of births (50%), the lowest proportion of miscarriages (29%) and the lowest proportion of ectopic pregnancies (8%). Those with coagulation sterilizations had the lowest proportion of births (32%) and the highest proportion of miscarriages (38%) and ectopic pregnancies (17%).

### Birth rates

**Figure 2:**
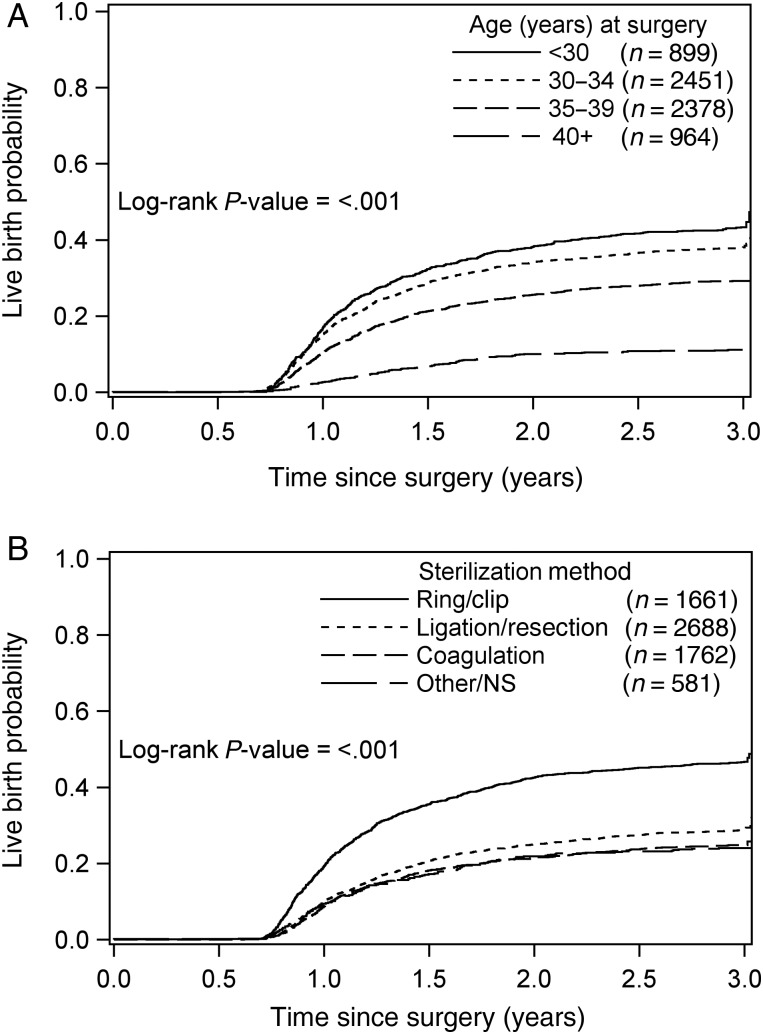
Kaplan–Meier estimates of cumulative live birth probabilities over 3 years by (**A**) age at surgery group and (**B**) sterilization method. NS, not specified.

In total, 2314 (35%) patients had at least one live birth during follow-up, and 598 who had not previously given birth had pregnancies ongoing when follow-up ended. The range was 1–8 live births. Table IV provides the raw proportions of women who ever gave birth during follow-up. Since not all women were followed for the same length of time and some pregnancies were ongoing at end of follow-up, these proportions can be viewed as lower bounds on the actual live birth proportions. The combined age and method-specific proportions vary substantially, from 63% for women under 30 years with tubal ring/clip sterilizations to 8% for women aged 40 years or older who had coagulation procedures. Estimated cumulative incidences over 3 years, by age at reversal surgery and by sterilization method, are shown in Fig. 2.

**Table IV:** Women with any live births during follow-up by age and sterilization method.

Sterilization method	Age (years)	Number of women	Any live births, *n* (%)
Ring/clip	<30	267	167 (63%)
	30–34	619	348 (56%)
	35–39	559	264 (47%)
	≥40	216	37 (17%)
Ligation/resection	<30	359	149 (41%)
	30–34	1017	388 (38%)
	35–39	952	278 (29%)
	≥40	360	43 (12%)
Coagulation	<30	216	75 (35%)
	30–34	648	230 (35%)
	35–39	654	159 (24%)
	≥40	244	19 (8%)
Other/NS	<30	57	30 (53%)
	30–34	167	61 (37%)
	35–39	213	50 (23%)
	≥40	144	16 (11%)

## Discussion

The desire for pregnancy after tubal sterilization has public health implications. More than 10 million women in the USA and 180 million women worldwide have had a sterilization procedure (EngenderHealth, 2002; Jones *et al.*, 2012). Among ever-married women in the USA aged 15–44 years in 1995, 26% reported having a tubal ligation (Mosher *et al.*, 2004); nearly 25% of women with an unreversed tubal ligation said they and/or their partner wanted the sterilization reversed (Chandra, 1998). In the US Collaborative Review of Sterilization, the percentage of women expressing regret after a tubal ligation was 20% for women aged 30 years or younger and 6% for women older than 30 years at the time sterilization. For women who were under 25 years of age at the time of sterilization, the regret rate was 40% (Hillis *et al.*, 1999).

Since microsurgical principles have been applied to tubal anastomosis surgery, studies consistently have reported success, whether performed by laparotomy or by laparoscopy with or without a surgical robot. Definitions of success have varied. The definitions include: tubal patency, pregnancy, uterine pregnancy, ongoing pregnancy, viable pregnancy, term pregnancy, delivery and birth. Studies have varied also from months to years in lengths of post-operative follow-up.

We found 116 studies of the effectiveness of tubal anastomosis for sterilization reversal in a comprehensive search of the English language literature. Almost all were retrospective clinical case series or cohort studies. Many of them have been cited in literature reviews (Gomel, 1977; Siegler *et al.*, 1985; Dubuisson and Chapron, 1998; Deffieux *et al.*, 2011). A complete reference list is available upon request to the communicating author.

Thirty-nine percent of the 116 studies were based on fewer than 30 patients with follow-up data. Thirty-seven percent of studies had 30–99 patients; 12% had 100–199 patients; 8% had 200–299 patients and 4% had 300 or more patients in the study population. The number of bilateral tubotubal anastomosis patients with follow-up data in all clinical studies ranged from 3 to 960; the median was 44.

The largest study by far, prior to our study, was not a clinical one. Payment data from the Quebec provincial health insurance system identified women who underwent sterilization reversal surgery from 1980 through 1999. No information was provided about methods of sterilization or sterilization reversal. Among 4369 women who had a reversal after sterilization, 61% became pregnant and 48% achieved a delivery (Trussell *et al.*, 2003). The study design identified only completed pregnancies in the study population, the effect of which is a higher proportion of deliveries and delivery rate than the proportion of births and crude birth rate in our study population. If births and ongoing pregnancies were combined into a single category in our study, the crude birth/ongoing pregnancy rate would be more similar to the delivery rate in the Quebec study.

Because the outcomes were unknown in 14% of pregnancies (the ongoing pregnancies), the birth rates shown in this report understate the actual birth rates in the study population. To conclude otherwise, one would have to assume that all ongoing pregnancies resulted in miscarriage or were ectopic. This assumption is improbable since many ongoing pregnancies had progressed beyond the first trimester at last patient contact.

Limitations of our study include probable underreporting of pregnancies based on patient-initiated reports; possible errors in the reporting of pregnancies or early miscarriages that may have been based solely on home pregnancy tests; and probable over-reporting of the diagnosis of ectopic pregnancies. Medical records of reported ectopic pregnancies, when made available, showed that many were pregnancies of unknown location that aborted spontaneously or after treatment with methotrexate. Some of these may have been uterine pregnancies misclassified as ectopic pregnancies. Nevertheless, if a patient reported a pregnancy to be ectopic—even if the diagnosis was suspected but unconfirmed—it was coded as such in the database. The factors (age and sterilization method) we identified as being associated with subsequent pregnancy should not be interpreted as being predictive, because in order to claim something to be a predictor, the association would need to be validated in an independent second prospectively studied group of representative patients.

As part of the informed consent process, patients were advised of the increased risk of ectopic pregnancy associated with tubal reparative surgery. Instructions were given for early pregnancy monitoring with serial hCG assays and transvaginal ultrasound examination. These instructions were given at the time of surgery and repeated each time a positive pregnancy test was reported. The instructions included the recommendation for pregnancy termination to reduce the possible risk of tubal rupture if no intrauterine gestation sac was observed by the time serum hCG reached 2500–3000 mIU/ml.

Our definition of tubotubal anastomosis included anastomosis of any distal segment to the interstitial tubal segment rather than defining these as tubocornual or tubouterine anastomosis, as in some previous studies. We also included patients in the study population who had additional surgical procedures performed at the time of tubotubal anastomosis. Additional procedures included uterine myomectomy, fimbrioplasty, ovarian cystectomy and adhesiolysis. These inclusions are additional factors that could result in differences in pregnancy statistics in our study from other patient populations.

Our literature search identified only one RCT of bilateral tubotubal anastomosis for female sterilization reversal (Rock *et al.*, 1984). This study found no significant differences in pregnancies or pregnancy outcomes using either loupe or microscope. Cochrane database reviews have found no RCTs comparing laparoscopic, open or robotic surgical techniques to reverse the effects of tubal sterilization procedures (George *et al.*, 2013), or comparing the effectiveness of tubal reversal surgery with IVF (Yossry *et al.*, 2006). In the absence of such RCTs, couples and their clinicians must rely on observational data.

The number of patients in this prospective study is considerably larger than the populations of all previous studies of bilateral tubotubal anastomosis. The large population size permits more accurate analysis of factors associated with pregnancy and pregnancy outcomes than has been possible previously.

## Conclusion

Bilateral tubotubal anastomosis is a successful outpatient procedure for women wanting fertility restored subsequent to tubal sterilization. Pregnancy and birth rates vary widely depending on age and prior sterilization method. These factors are known before sterilization reversal surgery and they were associated with long-term results with a high degree of accuracy when taken into account simultaneously. This study report will help inform patients and clinicians about tubal anastomosis as an effective alternative to IVF.

## Authors' roles

G.S.B. performed most of the surgeries and conducted the follow-up. J.M.T. and M.A.W. participated in analyses and interpretation of results.

## Funding

There was no external funding for this project. Funding to pay the Open Access publication charges for this article was provided by the Sterling Foundation, Chapel Hill, NC.

## Conflict of interest

We have no conflicts of interest to declare. G.S.B. started the practice but no longer owns or governs it.
